# Therapeutic Effects of Transplanted Exosomes Containing miR-29b to a Rat Model of Alzheimer’s Disease

**DOI:** 10.3389/fnins.2020.00564

**Published:** 2020-06-18

**Authors:** Yavar Jahangard, Hamideh Monfared, Arman Moradi, Meysam Zare, Javad Mirnajafi-Zadeh, Seyed Javad Mowla

**Affiliations:** ^1^Department of Molecular Genetics, Faculty of Biological Sciences, Tarbiat Modares University, Tehran, Iran; ^2^Department of Physiology, Faculty of Medical Sciences, Tarbiat Modares University, Tehran, Iran

**Keywords:** Alzheimer’s disease, miR-29, exosomes, *BACE1*, *BIM*

## Abstract

Alzheimer disease (AD) is a complex neurodegenerative disorder with no definite treatment. The expression of miR-29 family is significantly reduced in AD, suggesting a part for the family members in pathogenesis of the disease. The recent emergence of microRNA (miRNA)–based therapeutic approaches is emphasized on the efficiency of miRNA transfer to target cells. The endogenously made secretory vesicles could provide a biological vehicle for drug delivery. Characteristics such as small sizes, the ability to cross the blood–brain barrier, the specificity in binding to the right target cells, and most importantly the capacity to be engineered as drug carriers have made exosomes desirable vehicles to deliver genetic materials to the central nervous system. Here, we transfected rat bone marrow mesenchymal stem cells and HEK-293T cells (human embryonic kidney 293 cells) with recombinant expression vectors, carrying either mir-29a or mir-29b precursor sequences. A significant overexpression of miR-29 and downregulation of their targets genes, *BACE1* (β-site amyloid precursor protein cleaving enzyme 1) and *BIM* [Bcl−2 interacting mediator of cell death (BCL2-like 11)], were confirmed in the transfected cells. Then, we confirmed the packaging of miR-29 in exosomes secreted from the transfected cells. Finally, we investigated a possible therapeutic effect of the engineered exosomes to reduce the pathological effects of amyloid-β (Aβ) peptide in a rat model of AD. Aβ–treated model rats showed some deficits in spatial learning and memory. However, in animals injected with miR-29–containing exosomes at CA1 (cornu ammonis area), the aforementioned impairments were prevented. In conclusion, our findings provide a new approach for the packaging of miR-29 in exosomes and that the engineered exosomes might have a therapeutic potential in AD.

## Introduction

Alzheimer disease is a chronic neurodegenerative disorder that particularly affects the hippocampus, an area of the brain that is necessary for spatial memory formation (Mu and Gage, 2011). The disease disrupts intercellular communication, metabolism, and repair of healthy neurons. Globally, it is estimated that 50 million people actually suffer from dementia, of which 30 million to 35 million have AD (Fish et al., 2019). AD is hallmarked clinically by a progressive and gradual decline in cognitive function and neuropathologically by accumulation of amyloid-β (Aβ) peptides as β-amyloid plaques, the hyperphosphorylation of tau proteins, and neuronal and synaptic loss. Although there is still considerable debate about the cause of AD, the amyloid cascade hypothesis remains the best-defined and most studied mechanism for the disease (Selkoe, 2001; Hardy and Selkoe, 2002). The amyloid plaques are composed mainly of aggregated Aβ, which is derived from the proteolytic cleavage of β-amyloid precursor protein (APP) by the β-secretase (*BACE1*) and γ-secretase (*PSEN/presenilin*) enzymes (Chow et al., 2010). According to the amyloid cascade hypothesis, which is a key event in the initiation of AD, soluble amyloid β accumulation into toxic oligomers and amyloid plaques initiates a pathogenic cascade leading to accumulation of the hyperphosphorylated tau protein in neurofibrillary tangles, reduced numbers of synapses, death of neuronal cells, mitochondrial malfunction, and eventually loss of cognitive function (Drachman, 2014). Because of the complexity of AD, however, few medications were clinically administered to treat this disease. Limited percentage of approved medications and their poor effectiveness result in AD being incurable. Thus, numerous research has focused on AD’s pathology and etiology to overcome this difficulty (Scott and Barrett, 2007). Therefore, finding an effective therapeutic approach has clinical values.

MicroRNAs (miRNAs) are small non-coding RNAs that posttranscriptionally fine-tune the expression of their target genes (Femminella et al., 2015; Gebert and MacRae, 2018). The miR-29 family members have been shown to be downregulated in AD (Hébert et al., 2008; Shioya et al., 2010). The human miR-29 family consists of miR-29a, miR-29b, and miR-29c, where their sequences differ in only two or three bases. *BACE1*, one of the miR-29s family target genes, is overexpressed in AD, and its upregulation leads to amyloid plaque formation by increasing enzymatic digestion of APP and ultimately Aβ accumulation and senile plaque formation (Hébert et al., 2008; Zong et al., 2011). The miR-29 family is also reported as an important survival factor for neuronal cells, where its downregulation elevates proapoptotic (e.g., *Puma*, *Bim*, and *Bak*) as well as *voltage-*dependent anion channel (*VDAC1*) gene expressions (Kole et al., 2011; Roshan et al., 2014).

MicroRNA-based therapies can be achieved by either using miRNA antagonist or miRNA mimics to restore the normal miRNA expression and function. One of the major concerns in all forms of gene therapy approaches is how to deliver the recombinant DNA to its target cells. Recently, exosomes have been suggested as a natural vehicle for the targeted delivery of biological molecules and drugs to exact target cells (Lu et al., 2017; Mathiyalagan and Sahoo, 2017).

Exosomes are small vesicles with a size between 30 and 100 nm in diameter. They are involved in cellular communications of healthy cells, as well as some pathological processes such as neurological disorders, cancer, and cardiovascular diseases. Exosomes contain lipid, protein, mRNA, and small RNAs. Among small RNAs, miRNAs are highly enriched in exosomes. Therefore, exosomes can deliver their miRNA contents to the desired target cells and hence induce a change in the behavior of the cells. Consequently, miRNA delivery by exosomes to unhealthy cells has been recently considered as a potential therapeutic approach (Simons and Raposo, 2009; Andaloussi et al., 2013).

Here, we transfected bone marrow mesenchymal stem cells (r-BMSCs) and HEK-293T cells with miR-29a– and miR-29b–containing expression vectors and confirmed the packaging of the mature miRNAs within the exosomes released from the transfected cells. Transplantation of the miR-29 enriched exosomes into the hippocampal area of Aβ rat model of AD improved some deficits in spatial learning and memory of the animals.

## Materials and Methods

### Cell Cultivation

Mesenchymal stem cells were isolated from bone marrow of Wistar rats aged between 5 and 6 weeks as previously described (Soleimani and Nadri, 2008). r-BMSCs, human embryonic kidney cells 293 (HEK-293T), and glioblastoma cell line (U87) were cultured in Dulbecco modified Eagle medium (Gibco, Grand Island, NY, United States) supplemented with 10% fetal bovine serum (Gibco) and 1% penicillin/streptomycin (Bio Basic, Markham, Ontario, Canada) in a 37°C with 5% CO_2_ incubator.

### Transient and Stable Transfection of HEK-293T and r-BMSC

Hsa-mir-29a and hsa-mir-29b were cloned into Plex-Jred-puro (carrying red fluorescent protein and puromycin resistance gene) and pLenti-III-GFP (carrying GFP and puromycin resistance gene) vectors, respectively. The accuracy of cloning was confirmed by colony polymerase chain reaction (PCR), restriction enzyme digestion, and DNA sequencing. One day prior to transfection, HEK-293T cells (15,000 cells per cm^2^) and r-BMSCs (5,000 cells per cm^2^) were plated in six-well plates. The cells were transfected with 4 μg of plasmid, 3.7 μL of Lipofectamine 3000 (Invitrogen, Carlsbad, CA, United States), and 8 μL of p3000. After 18–24 h, transfected cells were analyzed by a fluorescent microscopy to determine the transfection efficiency. After 48 h of transfection and for generating stable cell line, the transfected cells were grown in the presence of 1.5 and 2.5 mg/mL of antibiotic puromycin (Sigma-Aldrich, Deisenhofen, Germany), as the optimum concentration for HEK-293 and r-BMSC, respectively. Then, the antibiotic concentration was slowly decreased.

### Exosome Isolation and Purification

Stable cell lines were cultured in BSA or fresh media that had been depleted of serum exosomes by ultracentrifugation at 100,000*g* for 3 h (Sokolova et al., 2011). Serum-free supernatants were collected every 2–3 days and centrifuged as previously described (Théry et al., 2006), with some modifications. Briefly, for eliminating residual cells, cellular debris, and vesicles bigger than 200 nm, conditioned media were centrifuged at 300*g* for 10 min, 2,000*g* for 30 min, 10,000*g* for 1 h, and 20,000*g* for 2 h. Finally, exosomes were pelleted at 100,000*g* for 70 min and were resuspended in 100 μL of phosphate-buffered saline (PBS) and stored at −20°C until use.

### Characterization of Exosomes

The morphology and size of the exosomes were examined by SEM. For this purpose, the exosomes were fixed with 3.5% glutaraldehyde (Sigma) in PBS for 20 min. The fixed exosomes were then dehydrated with ascending grades of ethanol. Then, the samples were left to dry at room temperature for 12 h and imaged using a SEM (Digital SEM, KYKYEM3200 (. The exosome sizes were measured by DLS. For DLS, ∼40 μg of exosomes was mixed with 420 μL of filtered PBS and sonicated for 20 min. The protein concentration contained in each exosome pellet was quantified via the Bradford assay.

### Exosomes Internalization Assays

Exosomes were labeled with PKH-26 (Sigma), according to the manufacturer’s instructions, with minor modifications. Briefly, 75 μg of exosome pellets was resuspended in 1 mL of diluent C. Separately, 2 μL of PKH-26 was mixed in 245 μL of diluent C. The exosome suspension was then added to the stain solution and incubated for 5 min. To stop the labeling reaction, an equal volume of 1% BSA was added. Then r-BMSC and U87 cells were seeded in a 24-well chamber slide, and 10 μg of PKH-26–labeled exosomes was added to the medium. After 2–4 h, recipient cells were fixed with 4% formaldehyde, and the nuclei of the cells were stained with 4,6-diamidino-2-phenylindole (DAPI; Sigma Aldrich, St. Louis, MO, United States). Finally, the cells were visualized using an inverted fluorescence microscope.

### RNA Extraction and Real-Time PCR

Total RNA was extracted from cells or exosomes using Trizol or Trizol LS (Invitrogen), respectively. After removing the brain from the skull, total RNA was isolated from hippocampal left and right cornu ammonis area (CA1) area. cDNA synthesis (Takara cDNA Synthesis Kit, Otsu, Japan) and real-time PCR for miRNAs and mRNAs detection were performed using SYBR Green reagent (Bio Fact, Daejeon, South Korea). The relative amount of miRNAs was normalized to that of 5S rRNA. *GAPDH* was also used as the internal control for *BIM* [Bcl−2 interacting mediator of cell death (BCL2-like 11)], *NAV3* and *BACE1* expression analysis. miR-29a, miR-29b, and their well-known targets including *NAV3*, *BACE1* and *BIM* were amplified using specific primers (Table 1). Finally, the real-time PCR data were analyzed by the comparative 2^–ΔΔ*Ct*^ method.

**TABLE 1:** List of primers used in this study.

**Name**	**Forward/Reverse**	**Sequence 5′→3′**
miR-29a PCR	Forward	ACAGACTCATTCCATTGTGC
	Reverse	CCACATGCAATTCAGGTCAG
miR-29b PCR	Forward	Purchased from Bon Yakhteh (Tehran-Iran)
	Reverse	
miR-29a cDNA	Forward	Purchased from Parsgenome (Tehran-Iran)
synthesis	Reverse	
miR-29b cDNA	Forward	Purchased from Parsgenome (Tehran-Iran)
synthesis	Reverse	
BIM	Forward	CAAGTCAACACAAACCCCAAGTC
	Reverse	GTCGTATGGAAGCCATTGCA
NAV3	Forward	CCGACTATTCCTTCCTTGC
	Reverse	TGCGTTTCCCATACATCTG
BACE-1	Forward	GGAGTACAACTATGACAAGAGC
	Reverse	CCATTAGGTAGAGTGAGATGAC
5s rRNA	Forward	GTCTACGGCCATACCACCCTG
	Reverse	AAAGCCTACAGCACCCGGTAT
GAPDH	Forward	ATGGGGAAGGTGAAGGTCG
	Reverse	GGGGTCATTGATGGCAACAATA

### Animals

Adult male Wistar rats with an average weight of 220–240 g (aged 6–7 weeks) were used. Animals had free access to water and food, under controlled temperature conditions (22°C–25°C) and light–dark cycle (12–12 h, lights on 7 AM). All experimental and animal care procedures were performed according to the ethical guidelines set by the Ethical Committee of Faculty of Medical Sciences, Tarbiat Modares University, which were completely in accordance with the NIH Guide for the Care and Use of Laboratory Animals.

Animals were randomly assigned into four groups as follows: (1) in PBS-injected (control) group, rats received PBS; (2) Aβ-injected group, in which 10 μg of Aβ was injected bilaterally to generate rat model of AD; (3) in exosome-miR29b group, Aβ plus exosomes derived from the stable r-BMSCs expressing mir-29b were injected simultaneously (exosomes loaded with miR-29b); and (4) in exosome-mock, Aβ plus exosomes derived from r-BMSC–expressing mock vector were injected simultaneously. Six rats were used in each experimental group.

### Drug Administration

To generate the animal model of AD, Aβ1–42 (Sigma) was injected bilaterally into the CA1 region of the dorsal hippocampus. Stock solutions of human β-amyloid 1–42 in 0.1 M PBS were prepared, as instructed by the manufacturer. For the intrahippocampal injection, rats were anesthetized with intraperitoneal ketamine (100 mg/kg) and xylazine (10 mg/kg), and under the stereotaxic surgery, two guide cannulas (21-gauge) were inserted into the hippocampus. Animals were recovered for 7 days after surgery. Injections were carried out over 5 min, using a 5-μL Hamilton syringe fitted with a 30-gauge blunt-tipped needle bilaterally into the hippocampal CA1 region (AP: 3.24, L: ±2.1, and DV: 2.2), according to the Atlas of Paxinos and Watson (2006).

The injection dose was determined based on our *in vitro* studies on U87 cells treated with engineered exosomes packed with miR-29b. We initially injected 10 and 20 μg of the engineered exosomes derived from HEK-293 into the CA1 region of the dorsal hippocampus and observed the positive recovery effects for 10 μg of miR-29b–enriched exosomes. Because the findings of HEK-293–derived exosomes were similar to those of r-BMSC–derived ones, we report here only the data obtained from r-BMSCs.

### Barnes Maze Test and Experimental Design

The Barnes maze protocol used in this study was based in part on a previously reported procedure (Morel et al., 2015; Pitts, 2018), with minor modifications (Supplementary Figure S1). The rats were examined in three phases: habituation, acquisition training, and acquisition probe tests. For habituation, rats were acquainted with the platform and the escape box 1 day before the first trial. During acquisition training, rats were tested with the escape box, four trials per day for 4 sequential days. Acquisition training consisted of placing a rat in the starting chamber for 2 min, the chamber was then raised, and the aversive stimuli (bright light) were switched on. Rats were allowed to freely explore the maze for 3 min, and the number of explorations per hole was recorded. To do the acquisition probe, rats underwent a 3-min probe trial 4 days after the final session of acquisition training. In this test, the escape box was removed from the apparatus. In order to eliminate olfactive clues from the maze and the boxes, the surfaces were cleaned with 10% alcohol solution, after each trial. The behavioral performances were recorded using a computer-linked video camera mounted 120 cm above the platform. Behavioral parameters were evaluated as previously described (Morel et al., 2015) including escape box latency, total errors, hole exploration frequency in the goal sector (GS), hole exploration frequency in the non-goal sector (NGS), goal sector preference (GS/NGS), target-seeking activity, path length, and mean velocity. All data were measured using EthoVision XT software (Noldus Information Technology, Wageningen, Netherlands).

### Open-Field Testing Procedure

Open-field test was performed to assay the motor activity and anxiety-related behavior. The animals were placed at the center of a black square arena (60 × 60 cm and 40-cm wall height) and allowed to explore the apparatus freely for 10 min. Finally, the total distance traveled and mean velocity were analyzed using a video-tracking software.

### Statistical Analysis

All data represent the mean ± SEM, and the statistically significant changes examined with *t* test, one-way and two-way ANOVA using GraphPad Prism v7 (GraphPad Software for Science Inc., San Diego, CA, United States). The experiments were conducted in duplicate with at least three biological repeats. The results were considered as significant when *p* < 0.05.

## Results

### Generating a Stable Cell Line Constitutively Secreting miR-29a and miR-29b

**FIGURE 1:**
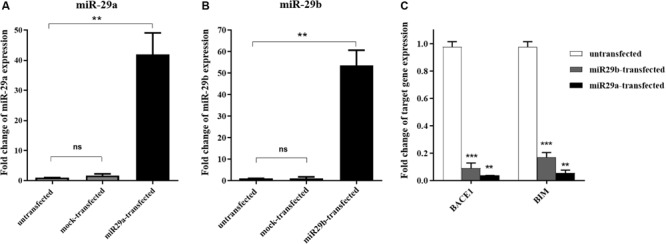
The transient expression of miR-29a and miR-29b in transfected HEK-293T cells. **(A,B)** A significant overexpression of miR-29a **(A)** and miR-29 **(B)** in HEK-293T cells, in comparison to the untransfected cells or cells transfected with mock vector. **(C)** Downregulation of miR-29 target genes (*BACE1* and *BIM*) in HEK-293T cells overexpressing mir-29a or miR-29b. All data are presented as the mean ± SEM. *< 0.05, ** < 0.01 and *** < 0.001, compared to the untransfected group.

We first examined the overexpression of miR-29a and miR-29b and their effects on some target genes in HEK-293T cells. For this purpose, precursors of miR-29a and miR-29b were transfected in aforementioned cell line. After 24 h of transfection, the morphology of the cells was examined under a fluorescent microscope. Stable cell line colonies were generated via antibiotic selection for at least 16 days (Supplementary Figure S2). After transient transfection, the expression levels of miR-29a and miR-29b were assessed using a real-time PCR approach. The results demonstrated that both miR-29a (Figure 1A) and miR-29b (Figure 1B) had been overexpressed in the transfected cells, compared to the mock-transfected or untransfected cells. To examine the functionality of the overexpressed miR-29a and miR-29b in HEK-293T cells, the expression of their well-known target genes, *BACE1* and *BIM*, was investigated. As expected, the overexpression of miR-29a or miR-29b downregulated the expression level of their target genes in transfected cells, in comparison to the untransfected cells (Figure 1C).

### Characterizing the Isolated Exosomes

**FIGURE 2:**
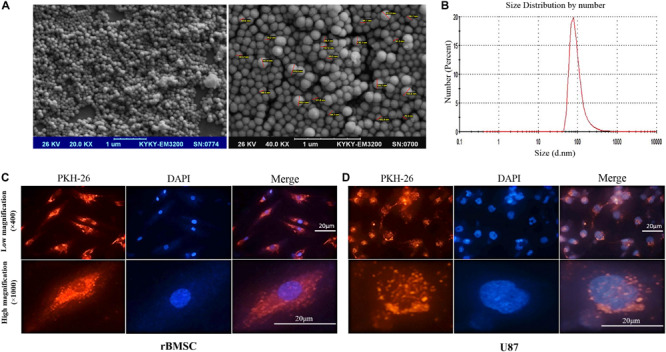
Characterizing exosomes and their internalization. **(A)** Scanning electron microscopy (SEM) analysis (magnifications of 20,000× and 40,000×) of exosomes demonstrated a spherical morphology for the isolated vesicles with a size range of 30 to 171 nm in diameter. **(B)** Dynamic light scattering image from isolated exosomes indicated a peak size at approximately 80 nm. **(C,D)** Internalization of cell-derived exosomes into U87 cells was confirmed via staining exosomes’ membrane with PKH-26 (showed in red). U87 cell nuclei staining is also evident by staining the cells with DAPI (showed in blue). Red fluorescence in the cytoplasm of the rat bone marrow stem cells and U87 cells indicated that significant amounts of the labeled exosomes were taken up by the cells.

To confirm the authenticity of the exosomes isolated from r-BMSC and HEK-293T cells media by differential centrifugation, we first measured the vesicle sizes, using DLS and SEM. SEM data demonstrated a spherical morphology and a size range of 50–171 nm in diameter for isolated exosomes (Figure 2A). In addition, exosome size measurement using DLS indicated a peak at approximately 80 nm (Figure 2B). To investigate the uptake of exosomes into r-BMSC and U87 cells, exosomes were labeled with fluorescent dye PKH-26, and the treated cells visualized using an inverted fluorescence microscope. Our data confirmed a successful uptake of the PKH-26–labeled exosomes by both cells (Figures 2C,D).

### Packaging miR-29a and miR-29b in Exosomes Secreted From the miR-29–Transfected Cells

**FIGURE 3:**
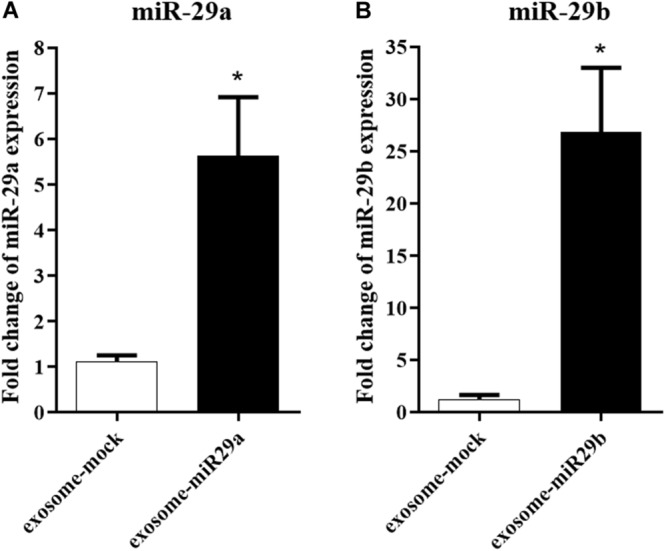
The packaging of miR-29a and miR-29b in exosomes isolated from HEK-293T cells. miR-29a **(A)** and miR-29b **(B)** levels were elevated in purified exosomes from HEK-293T cells, compared to the exosomes extracted from HEK-293T cells expressing mock vector, confirming miR-29a and miR-29b loading within exosomes. All data are presented as the mean ± SEM. * < 0.05 compared to the mock group.

To confirm a possible packaging of miR-29a and miR-29b in exosomes, we first assessed the presence of these miRNAs in the exosomes extracted from the stably transfected HEK-293T cells. Our data demonstrated that miR-29a (Figure 3A) and miR-29b (Figure 3B) were elevated approximately 5.6- and 26.8-fold, respectively, in the enriched exosomes isolated from HEK-293T cells stably expressing miR-29, compared to the cells stably expressing a mock vector (exosome-mock).

**FIGURE 4:**
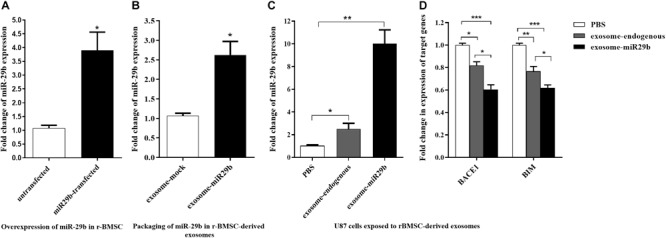
miR-29b packaging within the exosomes isolated from rat bone marrow stem cells (r-BMSCs) and its successful transfer to U87 cells. **(A)** Overexpression of miR-29b in r-BMSCs expressing mir-29b in comparison to the untransfected or r-BMSCs expressing the mock vector. **(B)** The level of miR-29b was elevated in exosomes derived from stable r-BMSC line exogenously expressing mir-29b, compared to exosomes extracted from r-BMSCs exogenously expressing mock vector. **(C)** Elevated level of miR-29b in U87 cells treated with r-BMSC–derived exosomes packed with miR-29b, compared to the cells treated with untransfected r-BMSC–derived exosomes (exosome-endogenous) or PBS. **(D)** Downregulation of *BACE1* and *BIM* in U87 cells treated with exosomes derived from stable r-BMSC line or exosomes derived from untransfected r-BMSCs, compared to the PBS-treated cells. All data are presented as the mean ± SEM. * < 0.05, ** < 0.01, and *** < 0.001.

Because miR-29a and miR-29b had the same expression pattern, seed sequence, and target genes, we continued our study with only miR-29b. Also, because of the inert therapeutic aspects of stem cells and their lower immunogenicity, we engineered r-BMSC for exosomes production. We first generated a stable r-BMSC line overexpressing miR-29b approximately 3.9-fold, compared to that of untransfected cells (Figure 4A). Moreover, the level of miR-29b was significantly elevated, approximately 2.6-fold, in purified exosomes obtained from r-BMSC stably expressing miR-29b (exosome-miR29b), compared to the stable r-BMSCs expressing mock vectors (Figure 4B).

To find out whether exosomes can transfer miR-29b to target cells, U87 cells were incubated with engineered exosomes containing miR-29b. Then, the expression of miR-29b and their target genes were quantified in treated cells. As expected, the expression of miR-29b was significantly elevated in U87 cells treated with exosomes packed with miR-29b, compared to the vehicle control (PBS) or exosomes derived from untransfected r-BMSC (exosome-endogenous). Furthermore, there was a significant difference in miR-29b expression in U87 cells treated with untransfected r-BMSC–derived exosomes than PBS (Figure 4C). To examine whether the exosome-mediated transfer of miR-29b is capable of regulating target genes, expression levels of *BIM* and *BACE1* were analyzed in treated U87 cells. Our data revealed that the exosomal transfer of miR-29b decreased the expression level of *BACE1* and *BIM* in exosomes-exposed cells (Figure 4D).

### Cognitive Impairment in Aβ-Treated Rats Was Partly Recovered by Engineered Exosomes

**FIGURE 5:**
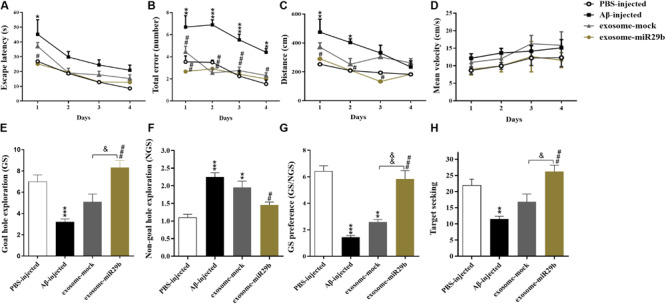
The transplantation of the engineered exosomes containing miR-29b could restore Aβ memory deficit. Amyloid-β–injected rats showed an increase in escape latency **(A)**, total errors **(B)**, distance **(C)**, and NGS **(F)**. In addition, Aβ microinjection decreased GS **(E)**, GS preference (GS explorations per hole/NGS explorations per hole) **(G)**, and target seeking **(H)** compared to the PBS-injected group. Injection of Aβ plus exosome-mock or engineered exosomes containing miR-29b (Aβ plus exosome-miR29b) reduced the Aβ-induced impairment in all of the mentioned parameters. No alterations of the mean velocity were observed in all groups **(D)**. Six rats were used in each experimental group. All data are presented as the mean ± SEM. *p < 0.05, ** < 0.01, ***p < 0.001, and ****p < 0.0001, in comparison to PBS-injected group. ^#^*p* < 0.05, ^#^#*p* < 0.01, ^#^##*p* < 0.001, in comparison to the Aβ-injected group. ^&^*p* < 0.05 and ^&^&*p* < 0.01 in comparison to exosome-mock group.

The Barnes maze test was used to assess spatial learning and memory in the rat model of AD. During the acquisition training, the escape latency time was prolonged in the Aβ-injected rats, compared to the PBS-injected (control) group. However, this increase was statistically significant only on the first training day (Figure 5A, *p* < 0.05). The total errors were significantly increased in the Aβ-injected group, compared to the control one, from the first to the fourth day of training (Figure 5B). The path length (distance) demonstrated a significant difference only on the first (*p* < 0.01) and second (*p* < 0.05) days of training, between the Aβ-injected and the control groups (Figure 5C). However, no difference in the mean velocity was observed in all experimental groups (Figure 5D, *p* > 0.05).

The probe test was performed to measure spatial memory after 24 h of the last training day. Results demonstrated that the GS (Figure 5E), the GS/NGS (Figure 5G), and the target seeking (Figure 5H) were significantly decreased, whereas the NGS (Figure 5F) was increased in the Aβ-injected group, compared to the control one. These findings imply that the injection of Aβ into the hippocampal CA1 region resulted in memory decline and deficits in spatial learning.

To examine the therapeutic effects of exosomes, we simultaneously transplanted either exosomes packed with miR-29b or mock vectors (exosomes + Aβ) into the CA1 region of the rat AD models. During the acquisition training, the total errors and the escape latency were reduced in the exosome-miR29b and exosome-mock groups, compared to the only Aβ-injected group (Figures 5A,B). The exosome-miR29b treatment significantly reduced the escape latency in the first retention training day, compared to the Aβ-injected group, but no significant difference was observed in the exosome-mock–injected group (Figure 5A). The total errors were decreased significantly throughout the training process in the exosome-miR29b and the exosome-mock groups, compared to the Aβ-injected group (Figure 5B). The path length demonstrated a significant difference from the first to third day of training between the exosome-miR29b and the Aβ-injected groups in the Barnes maze test (Figure 5C). Although the exosome-miR29b treatment had better effects on spatial memory improvement compared to the exosome-mock group, but there were no significant differences in the training days of these groups. There was also no significant difference between the exosome-miR29b and the exosome-mock groups compared to the PBS-injected group in all of the aforementioned parameters during the acquisition training.

During the probe test, the exosome-miR29b group showed a significantly higher exploratory activity for the GS than the rats injected with Aβ alone (Figure 5E). While the GS/NGS (Figure 5G) and the target-seeking activity (Figure 5H) were significantly increased, the exploratory frequency for the NGS (Figure 5F) decreased in the exosome-miR29b group, in comparison to the Aβ-injected group. Although the exosome-mock group showed a slight increase in GS, target-seeking activity, GS/NGS, as well as a decrease in the NGS compared to the Aβ-injected group, the observed differences were not statistically significant. Most importantly, significant differences were found in the probe test parameters between the exosome-miR29b and the exosome-mock–injected groups. Likewise, there was also no significant difference in the exosome-miR29b group compared to the PBS-injected group in all of the aforementioned parameters during the probe test. These results confirmed that the exosomes filled with miR-29b had a better therapeutic potential than the exosome-mock.

### Activity Monitoring by Open Field Test

**FIGURE 6:**
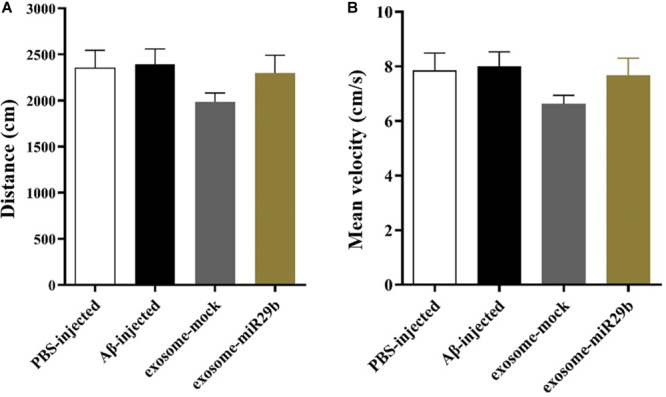
The distance traveled and mean velocity to reach the escape box assessed by the open field. There was also no significant difference in distance **(A)** and mean velocity **(B)** between groups in the open field test (between all groups). All data are presented as the mean ± SEM.

Path length and mean velocity are reference parameters for locomotion performance. No alterations of the distance (Figure 6A) and mean velocity (Figure 6B) in the open field arena were observed in all groups, indicating no motor deficits in this animal model.

### Transplanting Engineered Exosomes Upregulated the miR-29b and Downregulated Its Target Genes

**FIGURE 7:**
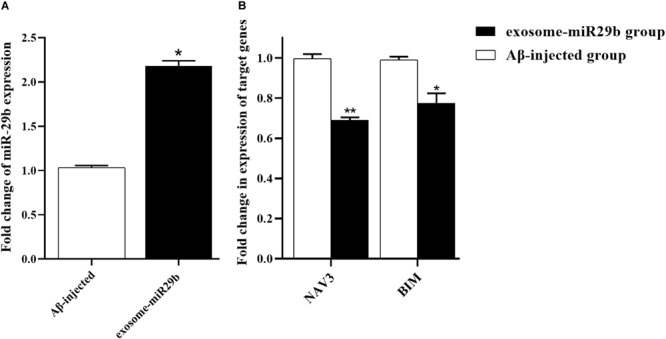
Transplantation of engineered exosomes caused an upregulation of miR-29b and downregulations of *NAV3* and *BIM*. Rats injected with Aβ plus engineered exosomes containing miR-29b (exosome-miR29b group) showed an evaluation in miR-29b level **(A)** and a decrease in expression levels of *NAV3* and *BIM* **(B)**, compared to the rats injected with only Aβ (Aβ-injected group). Six rats were used in each experimental group. All data are presented as the mean ± SEM. * < 0.05, ** < 0.01.

At the end of the behavioral tests, the animals were sacrificed, and their hippocampal’s left and right CA1 areas were rapidly removed and processed for RNA isolation. To determine whether miR-29 was upregulated and its main targets were downregulated in hippocampus areas injected with engineered exosomes, the expressions of miR-29b and *BIM* and *NAV3* genes were assessed. The results revealed that the level of miR-29b was significantly elevated in the rats injected with engineered exosomes containing miR-29b (exosome-miR29b group), compared to the rats injected with only Aβ (Aβ-injected group) (Figure 7A). Accordingly, the expression levels of *NAV3* and *BIM* were significantly decreased in the exosome-miR29b group compared to the Aβ-injected one (Figure 7B).

## Discussion

Alzheimer disease is the most common cause of serious cognitive problems, caused by dysregulation of the Aβ level or the hyperphosphorylation of tau proteins (Ballatore et al., 2007). Several miRNAs are differentially expressed in the brain of AD patients and dysregulate the expression of mRNAs with a part in AD pathogenesis (Cogswell et al., 2008; Nunez-Iglesias et al., 2010). A well-known candidate miRNA that could play a role in neuronal hemostasis is the miR-29 family. Based on the previous reports, the miR-29 family is downregulated in AD patients, causing a higher level of expression for their target, *BACE1* (Hébert et al., 2008; Shioya et al., 2010; Zong et al., 2011). Apart from their roles in amyloid plaque formation, the downregulation of miR-29b induces apoptosis in neuronal cells by elevating the levels of proapoptotic factors, such as *Bim*, *Bmf*, *Hrk*, *N-Bak*, and *Puma* (Kole et al., 2011). Moreover, the miR-29 family is shown to be an important regulator of neuronal cell survival in the brain, by controlling the expression level of *VDAC1.* Loss of miR-29 results in dysregulation of *VDAC1* and hence leads to neuronal cell death (Roshan et al., 2014).

Considering these previous findings, we made a hypothesis that administration of miR-29 can reduce the progress and/or the symptoms of AD. We also decided to deliver miR-29 to the hippocampal area via engineered exosomes packed with miR-29. We expected that using such natural intercellular communication vehicles would increase the success of our miRNA-based therapy of AD.

Because brain cells have limited regenerative potential, inhibiting unwanted apoptosis in the central nervous system has crucial importance. In addition, the miR-29 family plays an additional important role in synapse formation and synaptic plasticity by targeting *ARPC3* (*actin related protein 2/3 complex subunit 3*) (Lippi et al., 2011), as well as regulating axon guidance by controlling *NAV3* (*neuron navigator 3)* expression (Shioya et al., 2010; Zong et al., 2015). Altogether, it seems that the miR-29 family has vital roles in neuronal proliferation, death, and communication, as well as homeostasis. Therefore, elevating the tissue level of miR-29 in diseases such as AD, where the expression of miR-29 is diminished, could have therapeutic values.

There has been growing evidence that the miR-29 family could be a promising potential therapeutic target against AD. It was demonstrated that miR-29c promoted learning and memory behaviors in SAMP8 mice, which was associated with a decrease in the production of Aβ by targeting BACE1 and increasing the activity of PKA/CREB engaged in neuroprotection (Yang et al., 2015). The other study demonstrated that the recombinant pre-miR-29b using polyplexes allowed to decrease the hBACE1 and Aβ42 expression levels, highlighting the potential application of miR-29 in prognosis prediction and AD therapy (Pereira et al., 2016).

Despite its strong promise, challenges remain for the successful clinical application of ncRNA-based therapeutics. An ideally designed delivery system is crucial to transport ncRNAs to their site of action with minimal adverse effects *in vivo* (Chen et al., 2018). Exosomes are the most common choice because of the ability to cross physiologic barriers, generate low toxicity and immunogenicity, reduce interactions with non-target cells, and enhance cell entry and endosome escape and high structural and functional sustainability. Furthermore, there are many problems in designing exosome-based therapeutics. In particular, development and optimization methods for isolation and storage of exosome and improvement of therapeutic potential and delivery efficiency are required for fulfilling the therapeutic application of exosome (Yamashita et al., 2018).

As a natural delivery vehicle, exosomes hold a tremendous potential for the *in vivo* transfer of biological molecules to the desired cells. The presence of several mRNAs and miRNAs has been recently reported in exosomes. The exosomes and its genetic material contents can be taken up by neighboring (paracrine signaling) or distant (endocrine signaling) cells, where these genetics contents could deliver the signaling message to regulate physiological or pathological processes of the recipient cells (Valadi et al., 2007; Record et al., 2011). Recently, the therapeutic potential of exosomes isolated from stem cells has attracted some interest, as an alternative strategy to treat a number of diseases including cardiovascular, musculoskeletal, and immune systems and liver fibrosis diseases (Li et al., 2012; Rani et al., 2015; Wang et al., 2015; Gnecchi et al., 2016). One of the main obstacles in treating brain diseases is the presence of blood–brain barrier (BBB). Exosomes has recently emerged as one of the most promising approaches to non-invasive delivery of therapeutic agents to brain. There existed some reports that systemically injected exosomes can cross the BBB and reach the brain tissue, efficiently transferring administered siRNA-containing exosomes to the brain (Alvarez-Erviti et al., 2011; Liu et al., 2015). In a similar approach, it has been shown that intranasally administered exosomes can deliver curcumin to microglia or deliver miR-17 to inhibit brain tumor progression (Zhuang et al., 2011, 2016).

Here, we have examined a potential therapeutic value of the exosomes packed with miR-29b in a rat model of AD. To reduce immunogenicity, we employed exosomes isolated from rat bone marrow stromal cells, which has shown low immunogenicity in previous *in vivo* experiments (Alvarez-Erviti et al., 2011). To confirm the identity of the exosomes, exosome size measurement using DLS indicated a single peak at approximately 80-nm diameter. SEM data demonstrated 50- to 171-nm diameters of the extracted vesicles, corresponding to the size of the typical exosomes (Théry et al., 2009; György et al., 2011). The size of exosomes differs depending on the methodology used. Because of dehydration during sample preparation, the sizes estimated by electron microscopy are usually smaller, although for methodologies such as DLS, where the samples were not dehydrated, the sizes are larger (Soo et al., 2012). We also confirmed the presence of the specific exosomes markers on the surface of the extracted exosomes by Western blot assay in our previous study (Monfared et al., 2019).

We confirmed the packaging of miR-29a and miR-29b in exosomes isolated from the media of the transfected cells. We then examined whether the type of the employed cells could affect the packaging of miR-29 into exosomes. Our data confirmed the successful packaging of the miR-29 in both examined cell lines; however, the level of miR-29b in HEK-293T stable cell–derived exosomes was higher than r-BMSC stable cell–derived exosomes. Based on the packaging of miRNAs in engineered exosomes, we sought to investigate the exosomal transfer of miR-29b to target cells *in vitro*. As expected, our data confirmed an elevation in the level of miR-29b, concomitantly with a downregulation of its target genes (*BACE1* and *BIM*), in U87 cells treated with exosomes packed with miR-29b. Interestingly, there was a significant difference in miR-29b levels in U87 cells treated with non-engineered exosomes or only PBS injection, demonstrating that r-BMSC–derived exosomes naturally contain miR-29b.

Poor therapeutic effects of current treatments require new experimental models capable of mimicking AD pathology and then facilitate the exploration of therapeutic strategies and behavioral changes occurring in AD. Over the past decades, a variety of experimental models of AD, including transgenic and non-transgenic animals and *in vitro* models, have been developed (Drummond and Wisniewski, 2017). However, the experimental animal models currently available are not without limitations, and none of these models mimic all of the AD features. Choosing an experimental model relies on both the research aims and the study’s underlying goals. Admittedly, current Aβ-treated model rats do not provide a model of full-blown AD, because they are mostly deficient in tangle formation and neuronal loss, and synthetically deposited Aβ may not exhibit the same physical and biochemical characteristics as the amyloid found in AD. The Aβ-induced AD rats, on the other hand, are the most frequently used animal in experimental research because of the low cost, availability, and ease of manipulation (Drummond and Wisniewski, 2017; Kasza et al., 2017).

The hippocampus has a vital role in spatial learning and memory processes and affected during the early stages of AD. Aβ injection to induce AD in rats has been most often performed intracerebroventricularly; however, there are other reports that hippocampal injections of Aβ can rapidly impair hippocampal cognitive and metabolic processes (Geng et al., 2010; Pearson-Leary and McNay, 2012; Faucher et al., 2016). Our findings on the bilateral injection of Aβ into the hippocampus during the acquisition training revealed that escape latency was significantly elevated only on the first training day, the path length (distance) on the first and second days of training, and the total errors in the whole training days. Moreover, during the probe test, the GS, the GS/NGS, and the target seeking were significantly decreased, whereas the NGS was increased, in the Aβ-injected group. Also, the injection of engineered exosomes containing miR-29b into CA1 area could decrease the expression levels of *NAV3* and *BIM*, as previously reported (Kole et al., 2011; Zong et al., 2015). These results suggest that the injection of Aβ into the hippocampal CA1 region resulted in memory decline and deficits in spatial learning. Interestingly, the aforementioned impairments were entirely prevented in animals injected with miR-29–containing exosomes at CA1.

## Conclusion

Lack of inducing immune system, as well as their capacity to be engineered as drug carriers, has made exosomes desirable vehicles to deliver genetic materials to the central nervous system. Here, we have generated a rat model of AD with bilateral injection of Aβ into the hippocampus CA1 region, which caused some deficits in spatial learning and memory impairment. According to our data, injection of exosomes packed with miR-29b has protective effects against amyloid pathogenesis. Moreover, the engineered-exosomes showed a therapeutic effect on restoring the learning function of the hippocampus. Altogether, our findings suggest a new strategy for packaging miR-29 in exosomes and administering the engineered exosomes to prevent some memory deficits in an Aβ-induced AD model.

## Data Availability Statement

The datasets generated for this study are available on request to the corresponding author.

## Ethics Statement

The animal study was reviewed and approved by Ethical Committee of Faculty of Medical Sciences, Tarbiat Modares University. Written informed consent was obtained from the owners for the participation of their animals in this study.

## Author Contributions

YJ performed the experiments, analyzed data, and wrote the first draft of the manuscript. SM and JM-Z designed the study, interpret the data, and edited the final draft of the manuscript. HM, MZ, and AM helped in performing the experiments, interpreting the data, and critical read and commented on the manuscript.

## Conflict of Interest

The authors declare that the research was conducted in the absence of any commercial or financial relationships that could be construed as a potential conflict of interest.

## Supplementary Material

FIGURE S1 Design and the experimental protocol of the Barnes maze test. **(A)** The Barnes maze consisted of a dark, Plexiglas, circular disk 122 cm in diameter with 18 holes around the periphery. A black escape box or escape hole, (30 length × 12 width × 15 cm height) was located under one of the holes and the other holes are covered below with pages of the same color to make all the holes appearance the same. The escape hole is numbered as hole 0 (in a fixed position, relative to the cues) and the goal sector includes 0, 1, and −1, the remaining holes are non-goal sector. **(B)** A view of the Barnes maze apparatus. In the environment of the maze, various visual symptoms, including various geometric shapes, are exposed to direct animal vision. **(C)** 7 days after surgery, injection of PBS, Aβ or Aβ plus exosome were made. In 16th day, the open-field test was performed. Then, the rats were trained in the Barnes maze to escape into the escape box for 4 days (four trials per day) and the probe test was performed to measure spatial memory, 24 h after the last training day (14 days after injection of Aβ).

FIGURE S2 Transient and stable transfection of HEK-293T for generating of stable cell line colonies expressing mir-29a **(A)** and mir-29b **(B)**. 18–24 h after the transfection, cells were analyzed by a fluorescent microscope to determine the transfection efficiency. 48 h after transfection, stable cell line colonies were generated via antibiotic selection, by using 1.5 μg/ml Puromycin.

## Abbreviations

A β: Amyloid beta
AD: Alzheimer’s disease
BACE1: β -site amyloid precursor protein cleaving enzyme 1
BIM: Bcl − 2 interacting mediator of cell death (BCL2 like 11)
BSA: Bovine serum albumin
CA1: Cornu ammonis area 1
DLS: Dynamic light scattering
HEK-293T: Human embryonic kidney 293 cells
MiRNAs: MicroRNAs
r-BMSC: Rat bone marrow mesenchymal stem cell
SEM: Scanning electron microscopy.
