# Supplementary figures and images for: Therapeutic Effects of Transplanted Exosomes Containing miR-29b to a Rat Model of Alzheimer’s Disease
Source: Front Neurosci. 2020 Jun 18;14:564. doi: 10.3389/fnins.2020.00564 (PMC7314926; doi:10.3389/fnins.2020.00564)

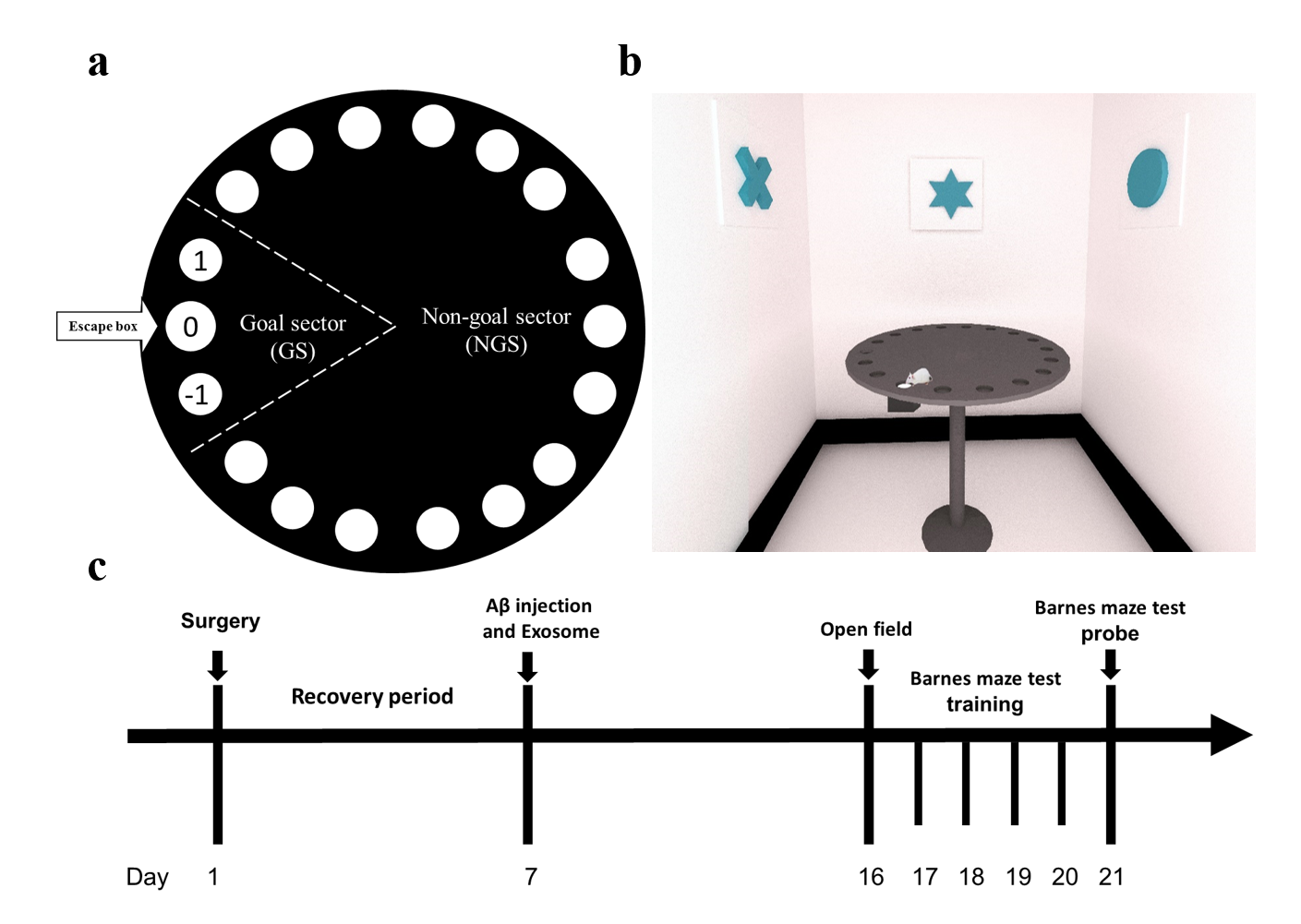

Supplement: FIGURE S1 — Design and the experimental protocol of the Barnes maze test. (A) The Barnes maze consisted of a dark, Plexiglas, circular disk 122 cm in diameter with 18 holes around the periphery. A black escape box or escape hole, (30 length × 12 width × 15 cm height) was located under one of the holes and the other holes are covered below with pages of the same color to make all the holes appearance the same. The escape hole is numbered as hole 0 (in a fixed position, relative to the cues) and the goal sector includes 0, 1, and −1, the remaining holes are non-goal sector. (B) A view of the Barnes maze apparatus. In the environment of the maze, various visual symptoms, including various geometric shapes, are exposed to direct animal vision. (C) 7 days after surgery, injection of PBS, Aβ or Aβ plus exosome were made. In 16th day, the open-field test was performed. Then, the rats were trained in the Barnes maze to escape into the escape box for 4 days (four trials per day) and the probe test was performed to measure spatial memory, 24 h after the last training day (14 days after injection of Aβ). [file Image_1.TIF]

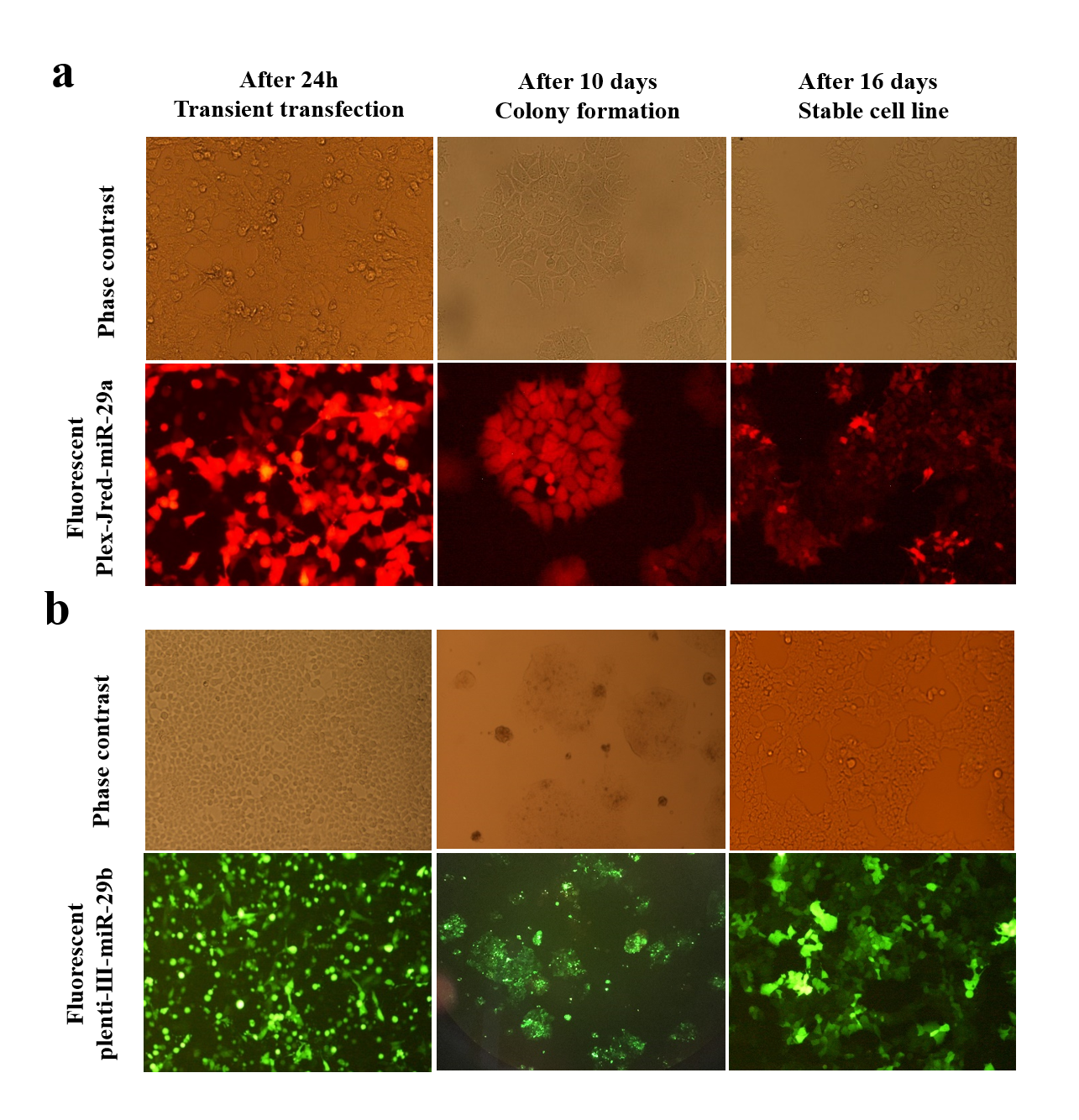

Supplement: FIGURE S2 — Transient and stable transfection of HEK-293T for generating of stable cell line colonies expressing mir-29a (A) and mir-29b (B). 18–24 h after the transfection, cells were analyzed by a fluorescent microscope to determine the transfection efficiency. 48 h after transfection, stable cell line colonies were generated via antibiotic selection, by using 1.5 μg/ml Puromycin. [file Image_2.TIF]
